# Tissue-Related Hypoxia Attenuates Proinflammatory Effects of Allogeneic PBMCs on Adipose-Derived Stromal Cells* In Vitro*

**DOI:** 10.1155/2016/4726267

**Published:** 2016-01-06

**Authors:** Polina I. Bobyleva, Elena R. Andreeva, Aleksandra N. Gornostaeva, Ludmila B. Buravkova

**Affiliations:** Institute of Biomedical Problems, Russian Academy of Sciences, Khoroshevskoye Shosse 76a, Moscow 123007, Russia

## Abstract

Human adipose tissue-stromal derived cells (ASCs) are considered a perspective tool for regenerative medicine. Depending on the application mode ASC/allogeneic immune cell interaction can occur in the systemic circulation under plenty high concentrations of O_2_ and in target tissues at lower O_2_ levels. Here we examined the effects of allogeneic PHA-stimulated peripheral blood mononuclear cells (PBMCs) on ASCs under ambient (20%) oxygen and “physiological” hypoxia (5% O_2_). As revealed with microarray analysis ASCs under 20% O_2_ were more affected by activated PBMCs, which was manifested in differential expression of more than 300 genes, whereas under 5% O_2_ only 140 genes were changed. Altered gene pattern was only partly overlapped at different O_2_ conditions. Under O_2_ ASCs retained their proliferative and differentiative capacities, mesenchymal phenotype, and intracellular organelle' state. ASCs were proinflammatory activated on transcription level that was confirmed by their ability to suppress activation and proliferation of mitogen-stimulated PBMCs. ASC/PBMCs interaction resulted in anti-inflammatory shift of paracrine mediators in conditioning medium with significant increase of immunosuppressive LIF level. Our data indicated that under both ambient and tissue-related O_2_ ASCs possessed immunosuppressive potential and maintained functional activity. Under “physiological” hypoxia ASCs were less susceptible to “priming” by allogeneic mitogen-activated PBMCs.

## 1. Introduction

The capacity of multipotent mesenchymal stromal cells (MSCs) to produce biologically active molecules, proliferate rapidly, and differentiate in several mesenchymal lineages as well as their immunosuppressive activity makes them very attractive tool for cell therapy and regenerative medicine [1]. Moreover, MSCs can be applied in an allogeneic setting due the absence/low expression of main histocompatibility molecules class II (MHC-II) and costimulatory molecules on the cell surface [2–4]. The molecular and cellular mechanisms underlying the MSC immunomodulation are currently being actively studied [5–9]. For the manifestation of immunosuppression, MSCs have to be activated (primed) [10–13]. This priming can be carried out by cytokines from activated lymphocytes, primarily, TNF-*α*, IFN-*γ*, and IL-1*β* [11]. After activation MSCs become visible to the immune system cells, such as the NK [14–16]. Thus, they are now MSCs considered more to be immune evasive than immunotolerant, which may affect their functions in allogeneic applications [17]. Most recent studies have focused on the immunomodulatory properties of MSCs, while their functions that are not directly related to immunosuppression are poorly explored. To date, the stability of the MSC mesenchymal phenotype (SSEA4, CD73, CD90, CD105, CD29, and CD44) after priming with proinflammatory cytokines [12, 18] and the retention of multilineage mesenchymal differentiation have been demonstrated [12]. These cytokines differentially modulate proliferative activity [12], the cytokine profile, and the migration of primed MSCs [11]. However, allogeneic MSCs being applied* in vivo* will be exposed not to certain cytokines but to a cocktail of proinflammatory mediators from immune cells. Besides, as shown previously, MSC/immune cells interaction is governed by the factors of local tissue microenvironment, where O_2_ level is the most important one [19]. Depending on the application mode, MSC/allogeneic immune cell interactions can occur in the systemic circulation under plenty high concentrations of O_2_ and in target tissues at much lower O_2_ levels. Therefore, here, we examined the paracrine effects of allogeneic activated peripheral blood mononuclear cells (PBMCs) under different O_2_ levels in the microenvironment on functional state and regeneration-related features of human adipose tissue-derived stromal cells (ASCs).

## 2. Materials and Methods

### 2.1. Isolation and Culture of Adipose Tissue-Derived Mesenchymal Stromal Cells (ASCs)

Adipose tissue samples were obtained in the frame of Scientific Agreement from multidisciplinary clinic “Souz” (Moscow, Russia) after elective liposuction procedures under local anesthesia from healthy patients with written informed consent. Adipose tissue was processed using guidelines specifically approved by Biomedicine Ethics Committee of Institute of Biomedical Problems, Russian Academy of Sciences (Physiology Section of the Russian Bioethics Committee, Russian Federation National Commission for UNESCO, Permit #314/МCK/09/03/13). Adipose stromal cells (ASCs) were isolated using standard method described by Zuk et al. with modifications by Buravkova et al. [20, 21]. Cells were expanded in *α*-MEM (22561-021, Gibco, Invitrogen, UK) with 50 U/mL penicillin-streptomycin (PanEco, Russia) and 10% fetal bovine serum (FBS) (sv30160.03, HyClone, USA) at either ambient O_2_ tension (20% CO_2_, CO_2_-incubator (Sanyo, Japan)) or under low O_2_ (5% O_2_) using a multigas incubator (Sanyo). Cells on the 2nd and 3rd passages were used in the experiments. ASCs expanded under different O_2_ were characterised [22] before use.

### 2.2. Isolation of Peripheral Blood Mononuclear Cells (PBMCs)

PBMCs were isolated by density gradient centrifugation (Histopaque 1077, Sigma-Aldrich, USA) of whole blood obtained from healthy volunteers after informed consent according to a standard protocol. Isolated cells were resuspended in RPMI-1640 (31870-025, Gibco) with penicillin-streptomycin and 5% heat-inactivated FBS. PBMCs were activated with 10 *μ*g/mL phytohaemagglutinin (PHA) (L8754-5MG, Sigma-Aldrich, USA).

### 2.3. Coculture of ASCs with PBMCs

ASCs were plated into the lower chamber of 6-well transwell plates (3412, Corning, USA, 0.4 *μ*m pore size). As ASCs reached a 70–80% monolayer, the medium was changed to RPMI-1640, 5% FBS, and 10 *μ*g/mL PHA. Activated PBMCs (10^6^/mL) were added to the upper chamber of the transwell. Cells were cocultured for 72 h at 20% and 5% O_2_.

### 2.4. ASC Viability

ASCs were trypsinised and the suspension was stained with Annexin V, FITC kit (PN IM3546, Beckman Coulter, USA). Live (Ann^−^/PI^−^), apoptotic (Ann^+^), and necrotic (Ann^−^PI^+^) cells were detected using flow cytometer (Epics XL, Beckman Coulter).

### 2.5. ASC Proliferation

To determine proliferation activity ASCs were counted in 5 fixed randomly selected view fields before experiment and in the same points after the coculture. Image acquisition was performed using Leica DMIL (Germany) and Nikon Eclipse TiU (Japan) microscopes equipped with digital cameras, and cells were counted using SigmaScan Pro 5.0 Image Analysis Software (SPSS Inc., USA). The proliferation activity was characterised as fold change of ASC number after experiment compared to Day 0.

### 2.6. Osteodifferentiation

After the experiment ASCs were cultured in complete *α*-MEM with osteoinductive supplement (100 nM dexamethasone, 10 mM sodium-b-glycerophosphate, and 0.05 mM ascorbic acid-2-phosphate) (Millipore, USA) for 3 weeks. Cells were fixed with 4% paraformaldehyde and stained with 40 mM alizarin red S solution (Sigma-Aldrich). Then matrix-bound dye was dissolved in 10% cetylpyridinium chloride (in 10 mM sodium phosphate buffer, pH7, w/v) and the OD was measured spectrometrically at 562 nm.

### 2.7. Mitochondria, Lysosomes, Endoplasmic Reticulum (ER), and Reactive Oxygen Species (ROS) Evaluation

Mitochondria, lysosomes, endoplasmic reticulum, and ROS were labeled with MitoTracker Red FM, LysoTracker Green, ERTracker, and H_2_DCFDA (Molecular probes, Invitrogen, USA), respectively, according to the manufacturer's protocol. ASCs then were detached with trypsin-EDTA and analyzed by flow cytometry (Epics XL).

### 2.8. Immunophenotypic Analysis

The immunophenotypic characterisation of ASCs was performed by flow cytometry (Epics XL) using the following monoclonal antibodies: CD45-phycoerythrin (PE), CD73-fluorescein isothiocyanate (FITC), CD90-FITC, CD105-PE, and CD54-PE (IO Test, Beckman Coulter). Trypsinised cells were incubated with the antibodies following the manufacturer's instructions.

### 2.9. Activation of PBMCs

To characterise the activation, PBMCs were stained with antibody against HLA-DR (PE) (IO Test, Beckman Coulter) following manufacturer's instructions and the share of HLA-DR-positive cells was estimated by flow cytometry.

### 2.10. Proliferation of PBMCs

Prior to experiment PBMCs were stained with carboxyfluorescein succinimidyl ester (CFSE, Invitrogen) according to standard protocol [23]. Proliferation rate was evaluated after flow cytometry by the proportional decrease of CFSE fluorescence in divided PBMCs.

### 2.11. Detection of Cytokines in Conditioned Medium

The conditioned medium (CM) was collected after coculture, centrifuged to remove cell debris, and stored at −80°C (low temperature freezer Sanyo). Cytokines were detected in CM using Human th1/th2 11plex FlowCytomix Multiplex Kit (BMS810FF, eBioscience, Bender MedSystems). FlowCytomix probes were analyzed using FaxCalibur cytometer (Becton Dickinson, USA) and FlowCytomix Pro software. LIF concentration in CM was estimated with Human ELISA Kit (ab100582, Abcam, USA).

### 2.12. RNA Isolation and Microarray Analysis

To perform gene expression profiling total RNA was isolated from ASCs monoculture and after 72 hrs of coculture with PBMCs at 20% and 5% O_2_. Cultured ASCs were detached with 0.05% trypsin-EDTA, washed with PBS, and preserved in RNAlater (Qiagen, USA) at −30°C. Before analysis, ASCs were washed off from the stabilizing agent and the standard procedure for RNA isolation using TRIZOL was applied (according to the manufacturer's protocol). RNA concentration was determined using a Nanodrop; then, 200 ng of RNA was amplified with Illumina TotalPrepTM RNA Amplification Kit (Ambion, USA). The amplified RNA was subsequently used for microarray hybridisation using Human-Ref-12 expression chip (Illumina, USA). The arrays were scanned and analysed using the Illumina Genome Studio v2009.2 software (Gene Expression Module v1.5.4, Illumina). The false discovery rate was controlled by adjusting *p* values by means of the Benjamini-Hochberg algorithm, followed by the performance of a Gene Set Enrichment Analysis and a one-tailed Fisher's exact test. The microarray data for 22184 genes were filtered by applying two criteria for significance: *p* < 0.05 and fold change (FC) > 2. To further analyse the data EASE v2.0 was used for the distribution of genes to Gene Ontology groups (biological function).

### 2.13. Statistical Analysis

Statistical analysis was performed using “Microsoft Excel 2010” and “Statistica 7.0” software packages. Differences were assessed by Mann-Whitney nonparametric test. All experiments were replicated, at least thrice, data are presented as mean ± SEM and *p* < 0.05 was considered to be statistically significant.

## 3. Results

### 3.1. Effects of PHA-Stimulated PBMCs on ASCs in Cell-Contact Independent Setting

**Figure 1:**
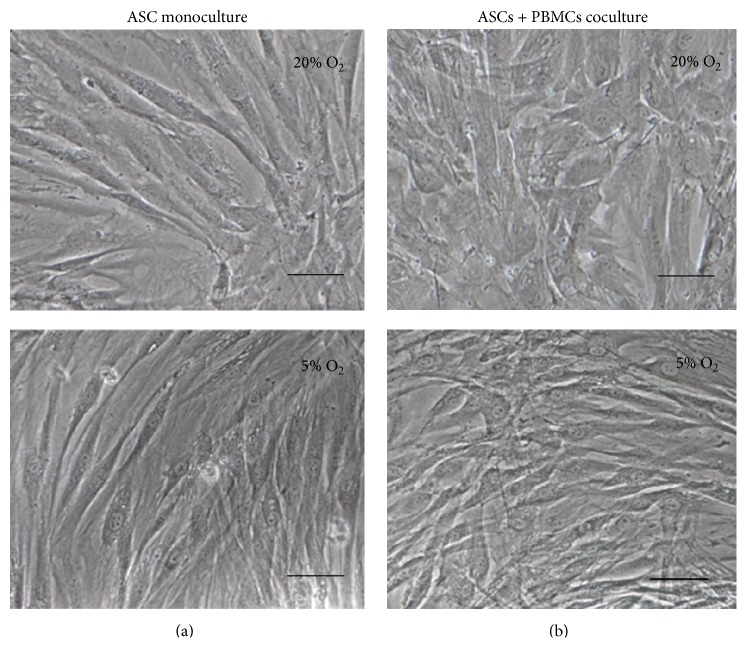
ASC morphology in monoculture and after transwell coculture with PHA-stimulated PBMCs under 20% and 5% O_2_. Phase contrast: bar 100 *μ*m.

Paracrine interaction with activated PBMCs during 72 hrs did not affect ASC morphology compared to ASC monoculture (Figures 1(a) and 1(b)).

**Figure 2:**
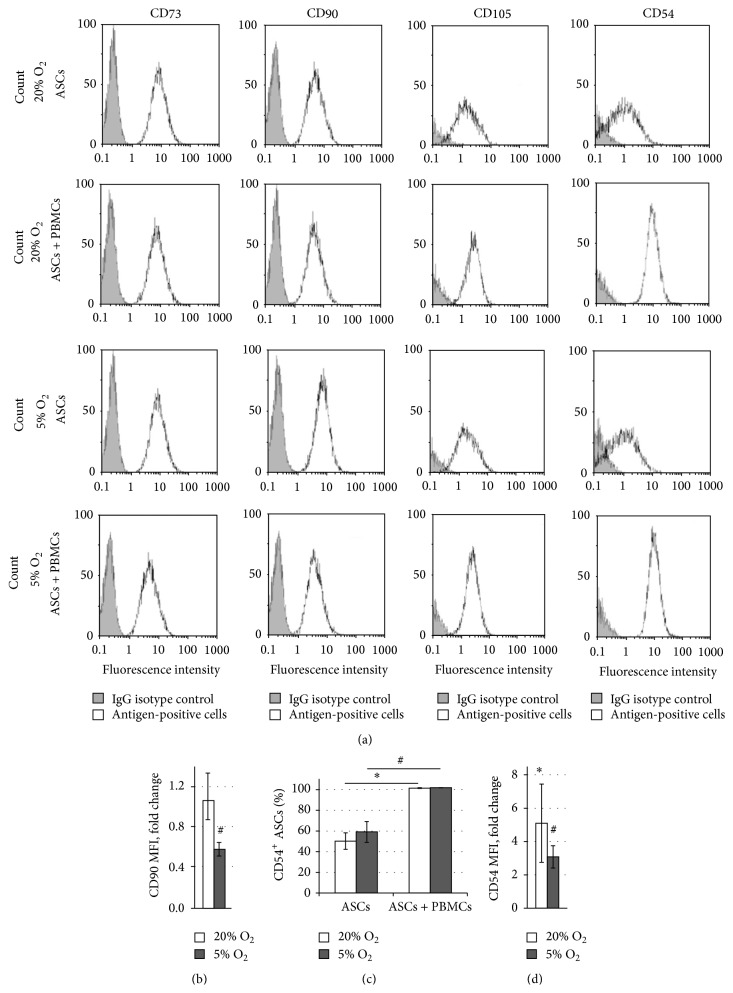
ASC immunophenotype after monoculture and transwell coculture with PHA-stimulated PBMCs under 20% and 5% O_2_. (a) Representative histograms of ASC surface marker expression. The white filled histograms indicate the positively stained cells while the grey filled histograms indicate the isotype-matched antibody controls. (b) CD90 mean fluorescence intensity (MFI) on cocultured ASCs versus ASCs in monoculture. (c) The proportion of CD54-positive ASCs in monoculture and transwell coculture with PBMCs. (d) CD54 MFI on cocultured ASCs versus ASCs in monoculture.  ^*∗*^Significant difference between monoculture and coculture at 20% O_2_ (*p* < 0.01).  ^#^Significant difference between monoculture and coculture at 5% O_2_ (*p* < 0.01).

Phenotyping of cocultured ASCs demonstrated that practically all of these cells were positive for CD73 (95.4 ÷ 98.7%), CD90 (95.4 ÷ 99.2%), and CD105 (94.0 ÷ 99.8%), which identified these ASCs as mesenchymal stem/stromal cells according to the recommendations of International Society of Cytotherapy [24] (Figure 2(a)). The mean fluorescence intensity (MFI), which characterised the number of antigen molecules on the ASC surface, did not change for CD73 or CD105. However, CD90 MFI of cocultured ASCs was significantly decreased under 5%  О_2_ (Figure 2(b)).

**Figure 3:**
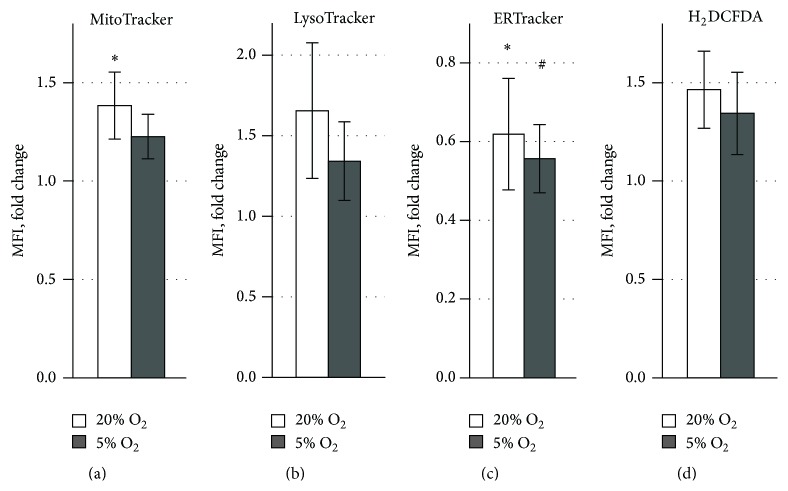
Cellular organelle state and ROS level in ASCs after coculture with PHA-stimulated PBMCs versus ASCs in monoculture. Transmembrane mitochondrial potential (a), lysosome activity (b), endoplasmic reticulum activity (c), and ROS production (d).  ^*∗*^Significant difference between monocultured and cocultured ASCs at 20% O_2_ (*p* < 0.01).  ^#^Significant difference between monocultured and cocultured ASCs at 5% O_2_ (*p* < 0.01).

Cellular organelles and ROS level were characterised in cocultured ASCs after staining with an appropriate fluorescent tracker (Figure 3). Under 20% O_2_ a significant increase of MitoTracker Red FM MFI was detected, displaying the elevated mitochondrial transmembrane potential (Figure 3(a)). No significant change in the MFI of LysoTracker Green was observed indicating the unaltered lysosome activity of ASCs after interaction with PBMCs (Figure 3(b)). Analysis of the ER compartment showed a significant decline of ER Green staining in cocultured ASCs both under 20% and 5% O_2_ (Figure 3(c)). The ROS level was not changed in ASCs after interaction with PBMCs regardless of O_2_ concentration (Figure 3(d)).

**Figure 4:**
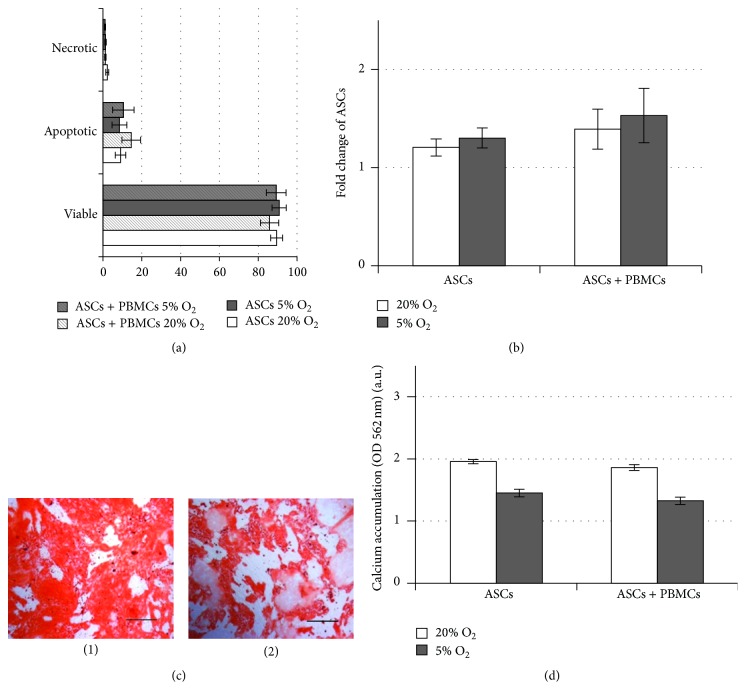
ASC functions after paracrine interaction with PHA-stimulated PBMCs. (a) ASC viability: percentage of viable, apoptotic, and necrotic cells in monoculture and after coculture during 72 h at 20% and 5% O_2_. (b) ASC proliferation: the change in cell number in monoculture and after coculture during 72 h at 20% and 5% O_2_. (c) Matrix mineralization of ASCs at 20% (1) and 5% O_2_ (2), alizarin red staining: bar 200 *μ*m. (d) Osteodifferentiation: matrix mineralization in monocultured and cocultured ASCs.  ^*∗*^Significant difference between ASC under 20% and 5% O_2_ in monoculture.  ^#^Significant difference between ASC under 20% and 5% O_2_ in coculture.

After paracrine interaction with allogeneic activated PBMCs, the viability of cocultured ASCs was high and did not differ from ASCs in monoculture (Figure 4(a)). Proliferative activity of ASCs was slightly increased during coculture with PBMCs (Figure 4(b)). ASC osteogenic capacity revealed by mineralised matrix production was not affected by interaction with PBMCs and was less marked under 5% O_2_ both in monoculture and after coculture with PBMCs (Figures 4(c) and 4(d)), confirming our recent findings [25].

After coculture, ASCs retained the immunomodulatory activity revealed in the suppression of T-cell activation (decrease in the share of HLA-DR-positive PBMCs) and proliferation (Table 1).

After interaction with PBMCs, practically all ASCs were CD54-positive, whereas, in monoculture, only half of ASCs expressed this antigen (Figure 2(c)). Moreover, the CD54 MFI was 5 times higher after coculture under 20% O_2_ and 2 times higher at 5% O_2_ (*p* < 0.05), indicating an increase in the number of ICAM-1 molecules per ASC (Figure 2(d)).

**Figure 5:**
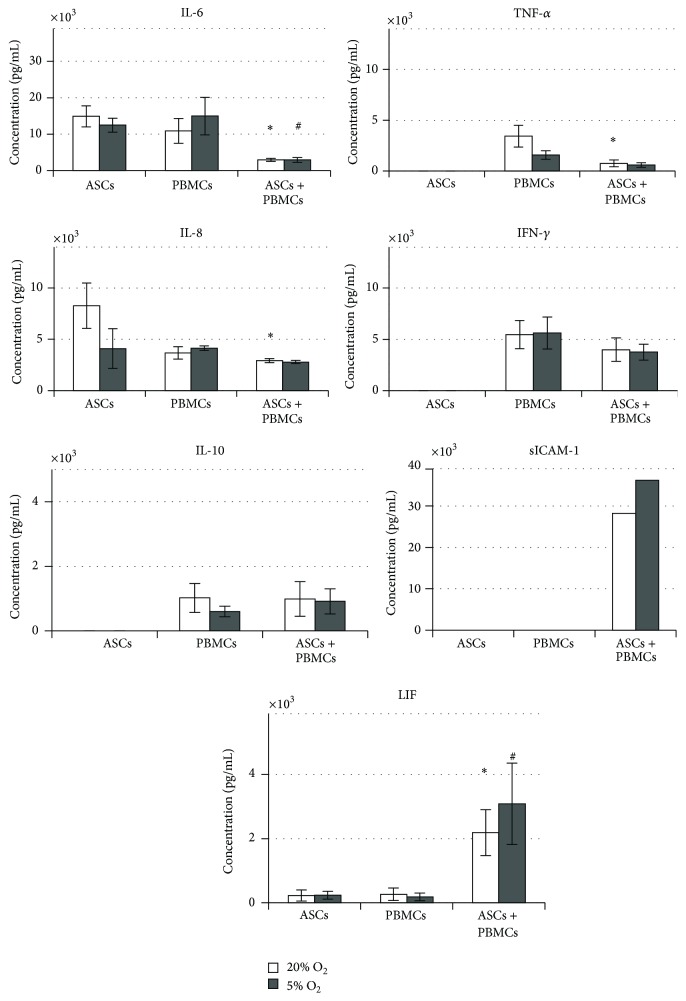
Concentration of soluble mediators in conditioned medium after 72 hrs of culture.  ^*∗*^Significant difference between ASC under 20% and 5% O_2_ in monoculture.  ^#^Significant difference between ASC under 20% and 5% O_2_ in coculture.

Paracrine mediators in conditioning medium (CM) of ASC and PBMC monocultures and after coculture were detected (Figure 5). IL-10, TNF-*α*, and IFN-*γ* were secreted only by activated PBMCs, whereas IL-6, IL-8, and LIF were detected in both monocultures. The level of cytokine secretion by ASCs and PBMCs did not depend on O_2_ concentration except of TNF-*α*, where the PBMCs synthesized significantly less of this chemokine under 5% O_2_. Cell-to-cell interactions affected paracrine profile. Besides membrane-bound ICAM1, soluble form of this adhesion molecule was detected after coculture (Figure 5). The reduction in the concentration of proinflammatory IL-6 and TNF-*α* was revealed (Figure 5). There were no significant changes in the level of IL-8, IL-10, and IFN-*γ* (Figure 5). Meanwhile, the level of LIF, which is known to be involved in ASC immunosuppression, was significantly increased after coculture (Figure 5). No effect of oxygen concentration on the production of soluble mediators was noted after coculture.

Thus, 72 hrs of indirect interaction with mitogen-stimulated allogeneic PBMCs did not lead to any alteration of ASC functions but provoked a significant shift in the profile of soluble mediators. It was of interest to assess how PBMCs affected the ASC transcriptional activity.

### 3.2. Effects of PHA-Stimulated PBMCs on ASC Gene Expression: Microarray Analysis

**Figure 6:**
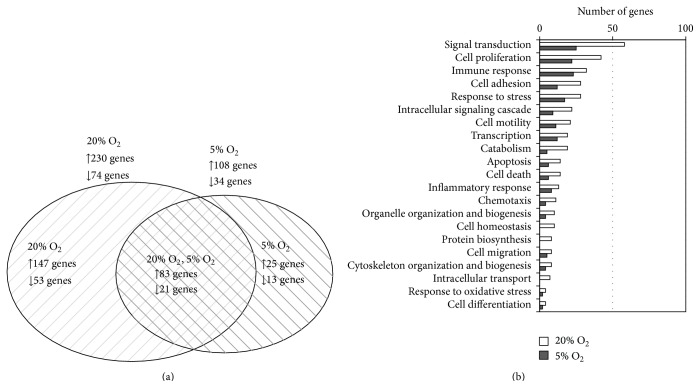
Characterisation of differential gene expression in ASCs after paracrine interaction with PHA-stimulated allogeneic PBMCs. (a) Venn diagram, showing the number of ASC genes commonly or differentially expressed at 20% and 5% O_2_. (b) The number of differentially expressed genes in Gene Ontology (GO) groups (biological function).

In this study, we identified genes that were differentially expressed in ASCs after interaction with activated PBMCs under different O_2_ conditions. Microarray analysis revealed significant change of ASC transcriptome profile: 304 genes at 20% O_2_ and 142 at 5% O_2_ were differentially expressed. In total, 104 genes were jointly changed both under 20% and 5% O_2_ (Figure 6(a)). Ranking according to Gene Ontology (GO) demonstrated that in groups of signal transduction, proliferation, immune response, cell adhesion, stress response, intracellular signal transduction, and cell motility 20 or more genes were differentially expressed under 20% O_2_. At 5% O_2_ the number of genes with altered expression in the same groups was lower. In some GO groups, the differentially expressed genes were detected only at 20% O_2_ (cell homeostasis, protein biosynthesis, and intracellular transport) (Figure 6(b)).

The alteration in the expression of certain most important ASC genes in different functional groups is summarized in Table 2. ASC proinflammatory activation-involved genes demonstrated the significant upregulation confirming “priming” of ASCs by PHA-stimulated PBMCs. The degree of this “priming” practically did not depend on O_2_ level in the milieu except for* TRAF3IP2* and* COLEC12*, whose upregulation was higher under 5% O_2_. Among the paracrine regulation-entailed molecules the significant upregulation was detected in СС-, CXC-, and IL-families:* CXCL1*,* CXCL12*,* CXCL5*,* CXCL6*,* CCL2 (MCP-1)*,* CCL5*,* IL11*,* IL1B*, and* IL8*. It is important that, except* MCP-1* and* IL-1β*, the overexpression was more evident under ambient 20% O_2_. Moreover,* CXCL1*,* CXCL5*, and* CXCL6*, whose expression increased more than ten- or hundred-fold under 20% O_2_, did not changed under 5% O_2_. Immunosuppression-associated genes displayed both up- and downregulation in different O_2_ conditions. So,* PTGIS* and* TGFBI* were negatively regulated under 20% O_2_, while displaying positive regulation under 5% O_2_. The data on* LIF* expression supported ELISA data presented above on enhanced production of this mediator under 5% O_2_. The group of genes, linked with extracellular matrix remodeling, showed significant increase of matrix metalloproteinase (*MMP1*,* MMP3*) gene transcription simultaneously with collagen upregulation (*COL7A1*). Expression of genes, encoding cell-matrix interaction-mediating molecules (*ITGA11*,* ITGB5*,* PODXL*, and* MFGE8*), was significantly downregulated after ASC/PBMCs interaction under 20% O_2_ and had no changes at 5% O_2_. Only* PDPN* was upregulated under both O_2_ concentrations. Migration-associated genes (*HAS1*,* SLIT2*) displayed enhanced transcription at 20% and 5% O_2_. Expression of proliferation-regulated genes (*CDKN3*,* CCNB2*) increased only under ambient O_2_, while* CDC20* and* MCM4* were upregulated at both O_2_ conditions.

In summary, the data on differential expression of ASC genes after interaction with activated allogeneic PBMCs demonstrated the significant shift in expression pattern that varied depending on O_2_ conditions.

## 4. Discussion

Cell-to-cell interaction is a dynamic process governed by local milieu. When responding to the microenvironmental challenges, cells not only modulate themselves but also regulate the properties of other cells in accordance with their new state. In the present study, we examined the effect of paracrine interaction of allogeneic activated PBMCs and ASCs on functions and the transcriptome of the latter under “physiological” hypoxia (at tissue-related O_2_). These effects have been addressed in two aspects: firstly, how does proinflammatory activation by allogeneic PBMCs affect the features (internal state) of ASCs themselves and, secondly, how do ASC functions change after interaction with activated immune cells?

Proinflammatory activation is considered one of the prerequisite for induction the immunomodulatory potential of MSCs [26]. It was shown that this effect is provided in the presence of activated immune cells, as well as certain inflammatory mediators (TNF-*α*, IFN-*γ*, IL-1*β*, and IL- 6). The effects of certain cytokines and of cocktail produced by activated PBMCs can differ significantly [11–13, 27]. So far, global differential gene expression analysis showed only a partial match for genes with altered expression in MSCs exposed to certain cytokines, combinations thereof, or a cocktail from the activated PBMCs. Wherein these certain cytokines stimulated IDO-mediated immunosuppression by MSCs, a cocktail from activated immune cells caused PGE_2_-immunosupression [27].

In this paper, gene microarray analysis showed that after 72 hours of ASC/PHA-activated allogeneic PBMC paracrine interaction the number of ASC genes with altered expression was more than twice of that under 20% O_2_ compared to 5% O_2_, and only half of them jointly changed under both O_2_ conditions. The upregulation of genes directly involved in “regenerative” ASC functions was detected in this “joint” group. There were genes regulating the cell cycle:* CDKN3* (cyclin-dependent kinase inhibitor),* CCNB2* (cyclin B),* CDC20* (cell cycle 20 homolog), and* MCM4*, the activity of which is related to the initiation of DNA replication. In addition, a negative regulator of cell proliferation,* CDKN2B*, was downregulated, which may also be associated with the increased proliferative activity of ASCs. Besides, the transcription of several other important molecules was upregulated in ASCs after interaction:* HAS1*, hyaluronan synthase, an enzyme that is responsible for the production of hyaluronic acid, which is produced in an active reparative processes allowing migration of stromal cells and vascular growth;* SLIT2*, a protein that positively regulates the production of other important membrane-bound proteoglycans, glypican, that provide adhesion and migration of cells; and* SPHK1*, sphingosine kinase, which phosphorylates sphingosine to sphingosine-1-phosphate, an important regulatory molecule for interaction, cell migration, and proliferation.

Our data demonstrated that ASC “priming” occurred upon interaction with activated PBMCs. Therefore, we detected the upregulation of TNF-*α* and INF-inducible protein genes (*TNFAIP3*,* IF44*,* GBP2*, etc.) that reflects ASC “pro-inflammatory” activation which is needed to stimulate the production of immunosuppressive factors. Indeed, ASCs upregulated the activity of* HLA-I*, which is consistent with previously described increase of the corresponding protein [28]. We also revealed the elevated transcription of* PTGS2*, prostaglandin G/H synthase and cyclooxygenase, which is known to activate the production of PGE_2_, one of the main factors inhibiting the proliferation of T-cells.

ASC/PBMC interaction significantly stimulated transcription of CC, CXC, and IL cytokine families' genes in ASCs. The most significant upregulation was demonstrated for pleiotropic chemokines (*CXCL5*,* CXCL1*,* IL8*,* IL1B*,* IL11*, and* LIF*). These molecules provide chemotaxis, intercellular and intracellular signaling, and regulation of cell proliferation including the autocrine path, participate in the immune response and inflammation, and are responsible for the regulation of blood leukocytes, such as monocytes (MCP-1), lymphocytes (CXCL12), granulocytes (CXCL6), and neutrophils (CXCL5, IL-8, and CXCL1). Furthermore, the participation of CXCL1 and CXCL5 in the stimulation of angiogenesis was shown, which is also very important for regeneration. Cytokine gene activity in some cases did not depend on the concentration of O_2_ (*CCL5*,* IL11*); for a number of genes (*CXCL5*,* CXCL6*, and* IL8*) there was significant upregulation at 20% O_2_, while for others this was detected at 5% O_2_ (CCL2,* IL1B*, and* LIF*).

Transcription of genes, associated with extracellular matrix remodeling, the most important function of MSCs, such as collagen (*COL7A1*) and matrix metalloproteinases (*MMP1*,* MMP3*), was upregulated after ASC/PBMC interaction. The expression of* MMP3* was increased by almost 300 times. Increased matrix-remodeling activity of ASCs was apparently associated with the differential expression of integrins (*ITGA11*,* ITGB3*, and* ITGB5*), which mediated cell-matrix interactions and decreased the expression of a negative regulator of migration, podokan (*PODN*).

Thereby, microarray analysis showed the upregulation of genes, where protein products provide increased proliferative activity of ASCs, and their ability to migrate due to the enhanced extracellular matrix remodeling. Under ambient O_2_ (20%) significantly more genes changed their expression after interaction with activated PBMCs compared to tissue-related O_2_ (5%). While the transcriptome changes were revealed at both O_2_ concentrations, the effect was less marked under 5% O_2_. It should be noted that some key genes as* IL-1b*,* LIF*, and* HAS1* were more significantly upregulated under 5% O_2_ compared to 20% O_2_.

Thus, it can be concluded that interaction with allogeneic activated PBMCs can awaken the “regenerative” potential of ASCs at least at the transcription level. Also, the significant shift in cytokine levels in coculture conditioned medium was detected compared to PBMC and ASC monocultures. It is known that MSC/ASCs in a proinflammatory microenvironment synthesize a variety of soluble mediators, which are of high importance for the implementation of regenerative processes such as EGF, FGF, PDGF, TGF-*β*, VEGF, HGF, IGF-1, Ang-1, KGF, and SDF-1 [26]. The production of immunosuppressive molecules by MSCs was observed after priming by cytokines from activated leukocytes, in particular, IFN-*γ* in combination with TNF-*α*, IL-1*α*, or IL-1*β* [26].

Here we compared the cytokine levels in ASC and PBMC monocultures with the cytokine profile after contact-independent coculture. For mediators secreted by PBMCs only, after cocultivation, a marked decrease in the concentration of TNF-*α* was demonstrated, whereas the level of IFN-*γ* and IL-10 was the same as in a monoculture. Both ASCs and PBMCs in monoculture synthesized IL-8. The concentration of IL-8 in the coculture was the same as in monocultures, which indicates a decrease in the production of this cytokine in at least one type of interacting cells. Based on the significant upregulation of* IL8* transcription that we found in ASCs after coculture with PBMCs, a substantial inhibition of IL-8 production by PBMCs should be supposed. In ASC and PBMC monocultures we detected two IL-6 superfamily mediators, IL-6 itself and LIF. The concentration of IL-6 was significantly lower after cocultivation compared to monocultures, which may indicate a decrease in IL-6 synthesis in ASCs as well as in PBMCs. Given the absence of transcriptional changes of IL-6 in ASCs, it is reasonable to assume that there is a significant inhibition of this interleukin production in PBMCs. In contrast, after coculture LIF increased significantly compared to monocultures. In addition, we noted upregulation of* LIF* expression in cocultured ASCs. Thus, taking into account the decreased concentration of proinflammatory mediators like TNF-*α* and IL-6 and the increased production of LIF, which is known to be one of the ASC immunosuppressive mediators [9, 26] the cytokine profile after ASC/PBMC interaction can be characterised as anti-inflammatory one.

In the present paper we have demonstrated that ASC/PBMC paracrine interaction was accompanied by an increase in the number of ICAM-1-expressing ASCs as well as ICAM-1 molecules per cell. Moreover, we also detected soluble sICAM-1 in conditioning medium after coculture. It is known that ICAM-1 plays an important role in leukocyte adhesion and migration in inflammation. Majumdar et al. [29] attributed such an increase in ICAM-1 expression to MSC activation by TNF-*α* and/or IFN-*γ*, produced by activated PBMCs. However, while Faßlrinner et al. [30] also found an increase in ICAM-1 expression after interaction of PBMCs and bone marrow MSCs, they demonstrated that blocking IFN-*γ*, TNF-*α* did not completely prevent the ICAM-1 elevation suggesting the existence of additional stimuli of ICAM-1 expression. Therefore, Ren et al. [31] revealed that IL-1 also induced ICAM-1 expression. The authors attributed the elevation in ICAM-1 with enhanced immunosuppressive activity of MSC due to the “concentration” of immune cells around MSCs. Our data on increased ICAM-1 MFI on ASCs after coculture with PBMCs support Ren et al.'s [31] findings. It is necessary to point out that under tissue-related O_2_ per cell increase of ICAM-1 was less marked. Besides ICAM-1 we also analysed the expression of another adhesion molecule Thy-1 (CD90), a counterreceptor for the leukocyte integrin Mac-1, which usually is considered as mesenchymal cell marker. Though practically all ASCs in mono- and coculture were CD90-positive, the per cell expression of Thy-1 was significantly decreased under tissue-related O_2_. As it was shown previously a decline in CD90 expression on MSCs can lead to the loss of immunosuppressive activity [32]. Although we did not detect the attenuation of ASC immunosuppression at “physiologic” hypoxia, the reported decrease of adhesive ICAM-1 and Thy-1 per cell expression may support the abovementioned assumption of weaker proinflammatory activation of ASCs under tissue O_2_.

ASC viability and intracellular compartment functions were not hampered after 72 hours of paracrine interaction with activated allogeneic PBMCs. ASCs retained their mesenchymal phenotype (CD73^+^, CD90^+^, CD105^+^, and CD45^−^) and the capacity to proliferate and osteodifferentiate, which indicates the preservation of ASC functions regardless of O_2_ concentration. Earlier, Crop et al., in 2010, showed no effect of proinflammatory activation on ASC osteo- and adipodifferentiation and even found an increase in ASC proliferation rate after 7 days of coculturing [27]. Increase of MSC proliferation after 6–8 days of stimulation with the activated lymphocyte paracrine cocktail was also demonstrated [30]. In this case, the key role apparently belonged not only to IFN-*γ*, whose action is usually associated with proinflammatory “priming” of MSCs, but by other cytokines as well [30, 33]. Preservation of ASC functions, regardless of the concentration of O_2_ in the microenvironment, has an important applied aspect in terms of realisation of allogeneic ASC regenerative potential* in vivo*.

“Physiological” hypoxia is one of the key parameters of the microenvironment, which can modulate the properties of the cells and their interaction. In several papers it has been convincingly shown that the expansion of MSCs under tissue-related O_2_ switched them to glycolytic energy metabolism, which was accompanied by an increase of proliferation and reduction of differentiation [22]. Furthermore, the upregulation of “stemness” genes was demonstrated [34–38]. These data suggest that at tissue-related O_2_ MSCs can possess properties of uncommitted progenitor cells. Accordingly, such changes may also affect the proinflammatory priming of MSCs. Thus, we demonstrated that under “physiological” hypoxia (tissue-related O_2_) ASCs were activated in a proinflammatory manner and retained their functions under ambient and tissue-related O_2_, though ASCs exhibited lesser proinflammatory activation at 5% O_2_. These data show that the net effect of ASC interactions with other cell types, considered in terms of the* in vivo* situation, depends on a complex set of factors, including the specific issues of tissue microenvironment.

## 5. Conclusions

Our data demonstrated that paracrine interaction of allogeneic mitogen-stimulated PBMCs and ASCs under ambient O_2_ as well as at “physiologic” hypoxia resulted in “priming” of ASCs with significant upregulation of genes involved in proinflammatory activation, immunosuppression, cell proliferation, cytokine regulation, and extracellular matrix remodeling. Under tissue-related O_2_ notably less genes were differentially expressed in ASCs compared to 20% O_2_. Meanwhile, under both O_2_ conditions ASCs retained their functions, immunosuppressive potential and provided the anti-inflammatory cytokine shift. These data are important in terms of allogeneic ASC implementation in cell therapy and regenerative medicine.

## Figures and Tables

**Table 1:** Activation and proliferation rate of PBMCs after coculture with ASCs.

	20% О_2_	5% О_2_
Activation (CD3^+^/HLA-DR^+^)	40.6 ± 6.5^*∗*^↓	48.6 ± 5.3^*∗*^↓
Proliferation rate	22 ± 5^*∗*^↓	15 ± 2^*∗*^↓

Data are presented as a percentage of changes, when effects in PHA-stimulated PBMC monoculture were considered as 100%. Mean ± SD of 5 independent experiments. ^*∗*^Significant difference from PBMC monoculture (*p* < 0.05).

**Table 2:** Summary of most important differentially expressed ASC genes after paracrine interaction with PHA-stimulated PBMCs under different O_2_ concentrations, as determined by microarray analysis.

Gene	Product	ASC + PBMCs versus ASCs, fold change	
		20% О_2_	5% О_2_
Proinflammatory activation			
*TRAF3IP2*	TRAF3 interacting protein 2	4.6	9.3
*IRAK3*	Interleukin-1 receptor-associated kinase 3	6.3	6.2
*IRAK2*	Interleukin-1 receptor-associated kinase 2	5.7	6.5
*TNFAIP3*	Tumor necrosis factor, alpha-induced protein 3	5.1	5.2
*IFI44*	Interferon-induced protein 44	5.8	6.8
*IFI6*	Interferon, alpha-inducible protein 6, transcript variant 2	11.1	10.6
*IFI6*	Interferon, alpha-inducible protein 6, transcript variant 3	4.4	6.3
*ISG15*	ISG15 ubiquitin-like modifier	4.3	4.6
*GBP2*	Guanylate binding protein 2, interferon-inducible	3.7	5.2
*IFI27*	Interferon, alpha-inducible protein 27	4.3	5.4
*HLA-H*	Major histocompatibility complex, class I, H	2.9	3.8
*COLEC12*	Collectin subfamily member 12	5.4	7.3

Paracrine regulation			
*CXCL1*	Chemokine (C-X-C motif) ligand 1 (melanoma growth stimulating activity, alpha)	46.3	1
*CXCL12*	Chemokine (C-X-C motif) ligand 12 (stromal cell-derived factor 1)	3.0	3.8
*CXCL5*	Chemokine (C-X-C motif) ligand 5 (CXCL5)	328.3	1
*CXCL6*	Chemokine (C-X-C motif) ligand 6 (granulocyte chemotactic protein 2)	200.7	1
*CCL2*	Chemokine (C-C motif) ligand 2 (monocyte chemotactic protein 1)	11.6	52.4
*CCL5*	Chemokine (C-C motif) ligand 5 (RANTES)	5.6	7.4
*IL11*	Interleukin 11	14.2	16.8
*IL1B*	Interleukin 1, beta	28.7	95.5
*IL8*	Interleukin 8	81.4	28.0

Immunosuppression			
*HLA-B*	Major histocompatibility complex, class I, B	4.6	5.1
*HLA-E*	Major histocompatibility complex, class I, F	2.7	1
*HLA-H*	Major histocompatibility complex, class I, H	2.9	3.8
*PTGIS*	Prostaglandin I2 (prostacyclin) synthase	0.13	2.0
*TGFBI*	Transforming growth factor, beta-induced	0.33	33.2
*LIF*	Leukemia inhibitory factor (cholinergic differentiation factor)	3.0	7.4
*PTGS2*	Prostaglandin-endoperoxide synthase 2 (prostaglandin G/H synthase and cyclooxygenase)	5.3	3.7

Extracellular matrix			
*COL12A1*	Collagen, type XII, alpha 1	0.2	1
*COL6A2*	Collagen, type VI, alpha 2	3.2	1
*COL7A1*	Collagen, type VII, alpha 1	6.5	7.3
*MMP1*	Metallopeptidase 1 (interstitial collagenase)	18.9	9.2
*MMP3*	Matrix metallopeptidase 3 (stromelysin 1, progelatinase)	266.6	317.6

Migration			
*PODN*	Podocan	0.1	0.1
*HAS1*	Hyaluronan synthase 2	12.8	8.1
*SLIT2*	Slit homolog 2	4.8	3.9
*SPHK1*	Sphingosine kinase 1	2.5	1

Cell-matrix interaction			
*ITGA11*	Integrin, alpha 11	0.2	1
*ITGB3*	Integrin, beta 3 (platelet glycoprotein IIIa, antigen CD61)	4.9	1
*ITGB5*	Integrin, beta 5	0.3	1
*PODXL*	Podocalyxin-like protein	0.1	1
*PDPN*	Podoplanin	4.6	4.2
*MFGE8*	Milk fat globule-EGF factor 8 protein (lactadherin)	0.2	1

Proliferation			
*CDKN3*	Cyclin-dependent kinase inhibitor 3	9.6	1
*CCNB2*	Cyclin B2	8.1	1
*CDC20*	Cell division cycle 20 homolog	6.0	17.5
*MCM4*	Minichromosome maintenance complex component 4	14.4	7.2
*CDKN2B*	Cyclin-dependent kinase inhibitor 2B	0.1	0.1

Fold changes are for comparison between ASCs in monoculture and coculture. Positive values indicate higher and negative values indicate lower expression in cocultured ASCs. *p* < 0.05.
